# Facilitating Development Research: Suggestions for Recruiting and Re-Recruiting Children and Families

**DOI:** 10.3389/fpsyg.2017.01525

**Published:** 2017-09-11

**Authors:** Lisa B. Hurwitz, Kelly L. Schmitt, Megan K. Olsen

**Affiliations:** ^1^Center on Media and Human Development, Northwestern University Evanston, IL, United States; ^2^Keiser University Ft. Lauderdale, FL, United States; ^3^KL Media Research Chicago, IL, United States

**Keywords:** recruitment, methods, protocol, developmental research, longitudinal

## Abstract

Recruiting children and families for research studies can be challenging, and re-recruiting former participants for longitudinal research can be even more difficult, especially when a study was not prospectively designed to encompass continuous data collection. In this article, we explain how researchers can set up initial studies to potentially facilitate later waves of data collection; locate former study participants using newer, often digital, tools; schedule families using recruitment phone/email/mail scripts that highlight the many benefits to continued study participation; and confirm appointments with other digital tools. We draw from prior methodological and longitudinal pieces to provide suggestions to others wishing to re-recruit families for longitudinal studies. In addition, we draw upon our own experience conducting a non-prospective longitudinal study 6 years after an educational intervention, in which we successfully re-located 122 (90%) and interviewed 101 of 136 (83% of the located sample and 74% of the full original sample) parents and their early adolescent children. Although the majority of participants were recruited via original contact information (especially phone numbers), using a range of strategies to recruit (e.g., search engines focused on contact information, social media) and motivate participation (e.g., multifaceted phone/email/mail scheduling scripts, flexibility in location and means of participation) yielded a more desirable sample size at relatively low costs.

## Introduction

Across developmental science subfields, researchers have called for more longitudinal research (e.g., Karmiloff-Smith, 1998; Meeus, 2016; Wartella et al., 2016). Such research provides compelling information about developmental trajectories, patterns, sequences, and pathways (Nicholson et al., 2002). However, conducting longitudinal research presents formidable challenges, chief among which is re-recruiting sizeable samples across waves of research (Ribisl et al., 1996; Cotter et al., 2002). Recruiting for cross-sectional studies with children is already quite demanding, requiring researchers to devise thoughtful sampling schemes, set up databases tracking contact with families, convince families to follow through and schedule study appointments, and confirm these appointments in a sensitive manner, all while working around families' busy schedules (Striano, 2016). In addition to these challenges, re-recruiting for longitudinal work presents the added difficulty of working with a finite pool of families who might become fatigued from participating in multiple waves of research or who might have moved or changed contact information over time (Agrawal et al., 1978; Cotter et al., 2002; Barakat-Haddad et al., 2009).

In the present paper, we first provide general background information on developmental scientists' interest and success in re-recruiting for longitudinal work and then describe our own recent foray re-recruiting families who participated in a brief Chicago-based 2010 educational intervention 6 years later for a follow-up study. We outline strategies for arranging initial studies to facilitate later waves of data collection, and locating and scheduling these families for new testing sessions after a lapse in time. We contextualize these strategies in light of previously shared wisdom in this area. Our primary aim is to inform the work of researchers conducting longitudinal research; however, many of the suggestions we share also could enhance cross-sectional participant management.

## Longitudinal research in developmental science

Numerous developmental scientists are attempting longitudinal research, recruiting participants for a study at one time, and then re-recruiting them for subsequent waves of research at later time points. Large-scale multi-disciplinary cohort and panel studies such as the Dunedin Multidisciplinary Health and Development Study (e.g., Silva, 1990; Stanton and Silva, 1992) and studies falling under the umbrella of the Early Childhood Longitudinal Study (ECLS) (e.g., Heatly et al., 2015) are the most renowned sort of longitudinal work in child development. Such studies require extensive and continuous planning and resources (Nicholson et al., 2002).

In addition to these large-scale efforts, many other scholars have conducted smaller scale follow-up studies, sometimes deciding to re-recruit families who participated in one (often lab-based) study after extensive periods without any contact. Researchers have conducted such longitudinal work across a host of domains, including spatial cognition (e.g., Lauer and Lourenco, 2016), language (e.g., Can et al., 2013), literacy and mathematics achievement (e.g., Krajewski and Schneider, 2009), temperament (e.g., Schwartz et al., 2003), personality (e.g., Harris et al., 2016), memory (e.g., Forman et al., 2011), self-regulation (e.g., Ayduk et al., 2000), health and physical development (e.g., Fein et al., 2014), mental health (e.g., Agrawal et al., 1978), and media use (e.g., Hanson, 2017). Similarly, researchers who evaluate interventions also have engaged in comparable, sometimes non-prospective, longitudinal research years after the conclusion of interventions. Such studies help assess the long-term impact of programs attempting to directly improve children's outcomes in areas such as education (e.g., Campbell et al., 2012) and health and fitness (e.g., Lazorick et al., 2014), and indirectly influence child outcomes by providing parents supports such as cash supplements (e.g., Huston et al., 2005) and drug counseling (e.g., Haggerty et al., 2008). Thus, a variety of developmental scholars are responding to calls for longitudinal research, even though in practicality there are often sizeable gaps in time between waves of research in these studies.

Without the resources of large-scale cohort and panel studies, researchers have met the challenges of re-recruiting original study participants with varied success. Some report it to be particularly challenging re-recruiting urban individuals with racial-ethnic minority and low socio-economic status (SES) backgrounds (e.g., Ribisl et al., 1996; Barakat-Haddad et al., 2009; Fein et al., 2014; Poehlmann-Tynan et al., 2015), older children (e.g., Cotter et al., 2002, 2005), and individuals with common names (e.g., Barakat-Haddad et al., 2009; Masson et al., 2013). Conversely, others suggest very high-SES families are especially challenging to re-recruit (Silva, 1990). In the scholarship reviewed in the preceding paragraph, researchers' re-recruitment rates, when reported or inferable across publications, ranged from less than 15% (Schwartz et al., 2003; Barakat-Haddad et al., 2009) to nearly 100% (Silva, 1990; Campbell et al., 2012). However, researchers often fail to report retention rates or information that can be used to infer these rates, or to even use consistent definitions of what they consider to be successful retention (e.g., locating a parent AND child vs. only locating the parent; Ribisl et al., 1996).

Along these lines, scant guidance exists for re-recruiting families for longitudinal research. A small number of developmental researchers have shared strategies to facilitate multiple rounds of data collection, foremost among which include (a) leveraging contact information participants provided at the beginning of the original study (e.g., Ayduk et al., 2000), (b) employing—often fee-based—databases to find updated contact information (e.g., Haggerty et al., 2008), and (c) providing increasing compensation across waves of data collection (e.g., Cotter et al., 2002), among other techniques described in more detail in the following pages. Nonetheless, typically, published studies in child development focus more on the measures administered across waves of data collection than the recruitment process. Much of the existing re-recruitment guidance exists in articles targeting general populations, with advice not always relevant to children and families (e.g., Ribisl et al., 1996), or in clinical- or practitioner-oriented journals, which often include strategies very specific to small, special populations of children (e.g., Masson et al., 2013). Our goal in the present manuscript is to extend this body of literature by outlining strategies for re-recruiting a wide variety of children and families for an assortment of potential follow-up studies. To do so, we report on our own recent experiences re-recruiting urban families for a longitudinal study by outlining the protocol we followed to re-recruit families, which evolved over the course of data collection (as is typical in longitudinal research; see Ribisl et al., 1996).

## Current context

We recently re-contacted families (original *N* = 136) who had participated in an 8-week educational computer game intervention when children were in preschool and kindergarten (*M* age at Time 1 = 5.24 years, *SD* = 0.71). The original study was approved by the University of Pennsylvania Institutional Review Board (IRB), and parents consented to allow their children to participate in the intervention and to fill out multiple rounds of questionnaires themselves. Children verbally assented for their own participation. We collected follow-up data from former study participants 6 years after the original intervention when children were in late elementary school (*M* age at Time 2 = 11.28 years, *SD* = 1.30). Time 2 data collection activities were approved by the Northwestern University IRB. This was not a prospective study that we planned from the outset of the original intervention; consequently, we had no intermediate contact with families between the original intervention and follow-up study. During Time 2 study sessions, parents consented to link their family's Time 1 and Time 2 data, to potentially be contacted for additional follow-up studies, and to provide updated questionnaire information; children provided written assent for their participation.

Our original sample lived in the city of Chicago, and was racially and ethnically diverse (28% Caucasian, 28% Hispanic, 20% African American, 24% Other or Mixed race), with approximately 60% of families with incomes below $40,000 and 45% receiving or eligible for government aid. The sample was slightly more racially diverse and less affluent than the general Chicago population (United States Census Bureau, 2016). Given the literature suggesting older children (Cotter et al., 2002), individuals from urban communities, and low-income or racial-ethnic minority backgrounds are particularly difficult to re-recruit (Ribisl et al., 1996), we recognized the challenge ahead of us in re-recruiting for this project.

We had modest financial but considerable human resources at our disposal. Our budget was a little over US$5,000 to recruit, travel to, and compensate participants. We also had part-time re-recruitment manpower from a faculty member, lab coordinator, doctoral student (who was collecting this data for her dissertation), and five undergraduate research assistants. The faculty member had conducted a similar longitudinal study nearly 20 years ago, before dramatic increases in the popularity of newer interpersonal communication tools, such as text messaging and social networking sites (Purcell, 2011; Duggan, 2013).

## Preparing initial studies to facilitate later waves of data collection

Researchers may not always know at the outset of an initial study if they will be able to conduct subsequent rounds of data collection, for a variety of reasons including tenuous funding (Nicholson et al., 2002). Likewise, the research literature may prompt new questions that were not under consideration as part of the original research, but which could be addressed by re-recruiting original participants. Indeed, this was the case for us. Even in these situations, there are several steps researchers can take to facilitate potential future rounds of data collection. First, researchers may partner with local organizations, including schools (Language Reading Research Consortium et al., 2016). These relationships can assist with both initial recruitment (Striano, 2016) and re-recruitment, depending on the initial language used in establishing said partnership and the nature of the study itself (see the discussion on locating participants below for more information).

Second, researchers can create a database with detailed participant contact information, including information such as participants' and their family members' full names and aliases (e.g., nicknames, maiden names), phone numbers, emails, mailing addresses, educational and employment information (if relevant), contact information for friends, neighbors and/or relatives, birthdates, plans to move or change names, physical descriptions, and favorite hangouts (Ribisl et al., 1996; Cotter et al., 2002; Haggerty et al., 2008; Barakat-Haddad et al., 2009; Masson et al., 2013). Researchers have the ethical responsibility to gain informed consent for the foreseeable uses of such information, as well as to ensure participants that providing such information is voluntary (National Association of the Education of Young Children, 2011). Researchers should also take reasonable efforts to store participants' personal information securely (e.g., password protected or encrypted files, stored on a non-wide area network (WAN) connected computer server). In addition, principal investigators should also limit access to sensitive information so as not to violate participant anonymity (Hartmann, 1992; American Educational Research Association, 2011). During our original data collection, we collected names, phone numbers, email addresses, and employment information for up to two parents; full names, birthdates, and preschool/kindergarten names for children; and mailing addresses for the entire family. This helped ensure high participation over the course of the original intervention, and, as described in more detail below, was invaluable to subsequent re-recruitment efforts.

Third, researchers can adopt a blanket policy of including an optional element on all consent forms seeking permission for future contact about later research opportunities (e.g., Masson et al., 2013). This easy addition to consent forms provides an opportunity for future outreach should the need arise. In the absence of this initial consent, unexpected follow-up contact may be perceived as a privacy violation, and prevent former participants from choosing to dedicate more time to a research project. Further, IRBs may have an ethical obligation to prevent non-consensual follow-up contact. Indeed, this seems to be a common standard across universities in the U.S. (e.g., J. Hecht, personal communication, July 1, 2016). It is important researchers take these initial safeguards, because children are special vulnerable populations (Hartmann, 1992).

Fourth, researchers should attempt to build rapport with both parents and children across research activities (Cotter et al., 2002) and establish clear branding via the use of university or other logos (Ribisl et al., 1996; Haggerty et al., 2008; Striano, 2016). Depending on the level of anonymity of the study, researchers could even establish a Facebook or similar social media group for participants. Having a blog or newsletter to update families about study findings also can be a way to maintain connections with families who have participated in prior studies. Further, providing information and resources that parents will find useful can help to keep them engaged (Ganz, 2016). In prospective studies, researchers sometimes send participants regular newsletters and birthday/holiday greetings (Ribisl et al., 1996; Language Reading Research Consortium et al., 2016). Such techniques can help ensure the research experience is positive for participants and help them identify with the research study (and perhaps sign-up for other cross-sectional studies with researchers even if a team does not attempt to follow up with a specific study).

Finally, researchers with sufficient foresight in certain sub-domains may wish to gain consent from parents to contact their family members, friends, neighbors or other professionals in their lives (e.g., clergy; case workers) for help locating them (i.e., participating parents and children) at a later point, and to have parents prepare notes for these individuals consenting for them to provide current family contact information (Ribisl et al., 1996; Passetti et al., 2000; Cotter et al., 2002). These permissions could later be leveraged to assist with locating study participants for later waves of data collection. As a caveat, this approach may not be appropriate for all topic areas. For example, seeking this information might be reasonable as part of a lengthy intervention but could be intrusive in a one-time lab session.

## Re-recruiting for longitudinal research

**Figure 1:**
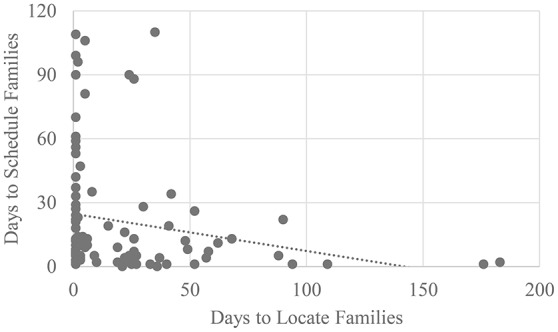
Scatterplot depicting the relation between the number of days it took to locate a family and the number of days to schedule said family once located (*r* = −0.20, *p* = 0.06).

In our discussion of re-recruitment for later waves of longitudinal research, we distinguish between *locating*, and actually *scheduling* families and engaging them in study *participation*. We consider a family to be *located* if a caregiver responds to re-recruitment attempts verbally or in writing, and acknowledges that we have identified the correct family, regardless of their level of interest in participating in the follow-up study (Haggerty et al., 2008). In contrast, we consider a family *to be scheduled and to have participated* in the study once they have arranged a time to meet with researchers and followed through with these appointments. Families who are difficult to locate are not necessarily difficult to schedule: In our study, the time we spent searching for a given family trended toward a negative correlation with the time it took to schedule said family once located (*r* = −0.20, *p* = 0.06; See Figure 1 for a graphic representation of this correlation and the online supplementary data table for information on the timelines and strategies employed in attempting to locate and schedule participants).

Although we located, scheduled, and engaged families in study participation simultaneously over a 6 month period, others have recommended dedicating some amount of time to locating participants prior to moving to the scheduling phase (Ribisl et al., 1996). That is, researchers may be well served by first locating as many families as they can and *then* scheduling them for follow-up appointments. Researchers may find certain families more time consuming to recruit than others, which in turn can prevent researchers from being able to speedily collect all data in a finite time period. When separating the locating and scheduling phases, families might receive a small monetary reward simply for providing updated contact information (followed eventually by a larger reward for participating and contributing data to subsequent waves of data collection; Ribisl et al., 1996). Additionally, more resources can be dedicated to families that researchers suspect might be challenging to locate based on prior interactions (Cotter et al., 2002), especially in light of research suggesting some families are consistently more challenging to re-recruit across multiple waves of research than others (Fein et al., 2014). In hindsight, we think this approach would have served us well and would be valuable for other developmental researchers, in that doing so might help researchers conduct all testing sessions succinctly once they have located most target participants. For example, it might have been wiser if we had dedicated all manpower resources to intensely focus on locating participants over a 3-month period, followed by a succinct and efficient 3-month testing period, rather than dividing our attention and manpower resources across these tasks over 6 months. Testing easier-to-locate children first and testing harder-to-locate children several months later might allow significant, potentially confounding developmental differences to arise.

In the remainder of this section, we outline strategies for locating and scheduling families. Our discussion of the former topic is particularly germane to those conducting follow-up studies, but our discussion of the latter topic includes strategies that also can be applied during the first round of data collection in a longitudinal study and during cross-sectional studies.

### Locating study participants

**Figure 2:**
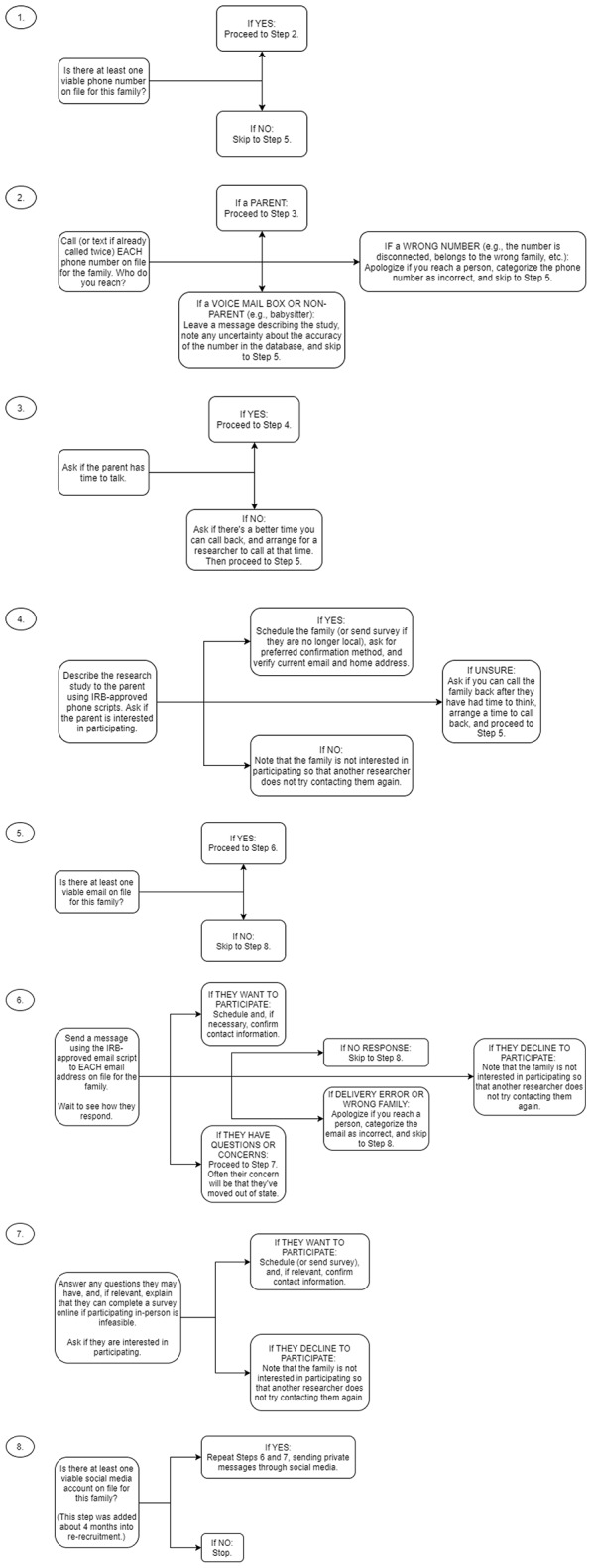
Schematic outlining the steps researchers took on a typical day when attempting to locate a family. Step 8 (outreach via social media) was added about 4 months into re-recruitment. Starting in month 3 of re-recruitment, researchers would forgo this series of steps 1 day per month, and instead spend a day preparing a large batch of recruitment letters for postal mail using mail merge.

We located 122 (90%) of the original participants in our study. We waited at least 2 weeks between attempts to reach the same family, and made efforts to initiate contact during a variety of times of day. Since our participating children were in late elementary school at Time 2, many lived in households where all caregivers had fulltime jobs, and accordingly, caregivers sometimes were more receptive to calls made outside of business hours (see Striano, 2016). However, other parents in our sample worked nontraditional jobs or hours, and thus were easier to reach midmorning. We engaged in a combination of phone calls and emails on a typical day of attempting to contact or reach out to a family, as described in more detail in Figure 2. Starting in the fourth month of re-recruitment, we also incorporated sending messages via social media into this routine. Also, on 1 day per month starting in the third month of re-recruitment, we prepared mass batches of letters to all unlocated families via mail merge, instead of following the usual procedure outlined in Figure 2. We acknowledge that we did not vary the order of steps described in Figure 2, and it is possible that researchers would see differing re-recruitment rates (either by contact platform or overall) if they were to conduct these steps in a different order.

On average, it took us 22.9 days (*SD* = 35.04) or about 2–3 days of outreach attempts from the time we first tried to locate families via any means to when we successfully located them. As is evident by the large standard deviation, there was considerable variability in the ease of locating families: We located 67 families (55% of located families) in less than 1 week (i.e., following one contact attempt day), 25 families (20% of located families) within 1 month (i.e., following about three contact attempt days), and the remaining 30 families (25% of located families) after 1 month. As explained above, we may have been able to locate some families somewhat more rapidly had we not also been collecting data simultaneously and been able to dedicate more manpower resources to location. Based on Time 1 data, located families had higher incomes [*t*_(39.14)_ = 4.82, *p* < 0.001], were more likely to be Caucasian, and were less likely to be of Other/Mixed race-ethnicity than families we failed to locate [$\chi_{\left(3,\;N\;=\;136\right)}^{2}$ = 11.13, *p* = 0.01, with significant pairwise comparisons for Caucasian and Other/Mixed races]. Otherwise, there were no differences between located and non-located families.

As we describe in more detail below and in Table 1, we located families using the contact information they provided at Time 1, and searching for updated contact information via free and paid tools. We also outline location strategies we did not believe would be successful for our study but that other researchers may find useful.

**Table 1:** Techniques for locating and scheduling participants (*N* = 136 original study participants).

**Contact method**	**Located**			**Participated**			**Days taken to locate via successful method**		**Days between location and participation**	
	***n***	**% of original participants**	**% of located participants**	***n***	**% of original participants**	**% of participating participants**	***M***	***SD***	***M***	***SD***
Original contact info.	100	74	82	85	63	84	11.36	21.62	22.15	28.26
Phone	75	55	62	61	45	60	11.35	23.62	23.18	29.75
Call	71	52	59	58	43	57	11.93	24.15	23.73	30.36
Text	4	3	3	3	2	3	1.00	0.00	12.33	8.50
Email	24	18	20	24	18	24	11.58	14.71	19.39	24.10
Mail	1	1	1	0	0	0	7.00			
Contact info. via free sources	19	14	16	14	10	14	14.00	13.78	18.50	27.76
Phone (calls)	9	7	7	8	6	8	12.22	14.29	12.05	10.20
Email	6	4	5	4	3	4	14.00	11.10	38.00	48.39
New email	4	3	3	2	2	2	22.33	5.86	17.50	6.36
Gmail based on original	2	2	2	2	2	2	1.00	0.00	58.50	72.83
email	4	3	3	2	2	2	18.00	18.96	3.50	2.12
Social media (Facebook)										
Contact info. via paid sources	3	2	3	2	2	2	5.33	3.79	1.50	0.71
Phone (calls)	1	1	1	0	0	0	1.00			
Mail	2	2	2	2	2	2	7.00	9.00	1.50	0.71

*Number of families who first responded to recruitment attempts via each contact method, percentage located of the original sample (N = 136) and percentage of located sample (n = 122) responsive to each method, number of families who participated in the follow-up after responding to each contact method, percentage of the original sample (N = 136) and percentage of participating sample (n = 101) who participated after responding to each contact method, days taken to locate via successful method [the average number of days between when researchers first attempted to contact families and when they finally located them, categorized by the method that ultimately provided to be successful for location (where the day initially contacted = day 1)], the standard deviation on that number, days between location and participation (the average number of days between when researchers first located a family and when the family participated in the study per method), and the standard deviation on that number*.

#### Originally provided contact information

We used originally provided contact information to attempt to call, text, and/or email all 136 parents, and in the third month of data collection, sent traditional letters to 33 parents who had not already been located (e.g., Ayduk et al., 2000; Anderson et al., 2001). In contrast to other studies, where researchers only saw moderate success leveraging originally provided contact information (e.g., Haggerty et al., 2008), we found that reaching out to caregivers using the information they provided in the original study was by far the most successful location strategy we employed. Traditional phone calls seemed to be particularly fruitful, but it is possible that our outreach protocol, which began with phone calls, may have influenced this result. Although many of the participants in the original study had moved from the specific homes they lived in at Time 1, most were still living in the greater Chicago metropolitan area and had retained their cell phone number or email address. Had we exclusively relied on original contact information, we still would have located over 70% of the original participants, which some would argue is an acceptable retention rate (Ribisl et al., 1996). We thus would have saved ourselves many manpower hours attempting the strategies outlined below. Growing reliance on cell phones means that individuals, especially in urban areas such as where we collected our data and especially for higher-SES populations, are increasingly maintaining the same phone number even after they relocate (Dost and McGeeney, 2016). This may in part explain some of our success using several-years-old phone numbers to re-recruit relative to prior research conducted before increased cell phone penetration.

#### Searching for participants via free online tools

We employed multiple free strategies to ascertain updated contact information in all cases where originally provided information was potentially outdated. First, we leveraged a variety of free online search engines and databases (e.g., Passetti et al., 2000; Cotter et al., 2002) to search for updated phone numbers, emails, and postal addresses for 58 families. Again somewhat contrasting with prior work, where free resources only yielded a small amount of accurate contact information (e.g., Haggerty et al., 2008; Masson et al., 2013), this was the most successful search avenue for us after exhausting original contact information. Mirroring recent similar studies (Cotter et al., 2002; Haggerty et al., 2008; Barakat-Haddad et al., 2009; Fein et al., 2014), we utilized general search engines such as Google, online databases known for contact information (e.g., the National Change of Address database) and people-centric search engines (i.e., search engines designed to scrape the Internet just for publicly available contact information). We found pipl.com, zabasearch.com, Whitepages.com, and ReferenceUSA.com to yield fruitful results, although we caution researchers that there is high turnover across such websites (for a similar warning, see Masson et al., 2013). Several websites recommended in prior, relatively recent methods pieces were no longer live during our data collection (e.g., dat+.us; Haggerty et al., 2008). We suspect our success with free resources may in part be attributable to the large volumes of contact information some people now post online through personal websites, public social network profiles, and the like (Rainie et al., 2013), and to newer blogs and articles showcasing high quality free people-centric search engines (e.g., Boswell, 2007). It also may be that the algorithms these services use have improved over time.

Second, we tested plausible alternate email addresses for 14 parents. Specifically, we located a small number of families by pairing the local part of email addresses parents had provided at Time 1 with Gmail domains (e.g., If a mother indicated her email address was something like sparklequeen78@sbcglobal.net at Time 1, we attempted to contact her at Time 2 using sparklequeen78@gmail.com). We noticed early into recruitment, before we began implementing this strategy, that several parents had changed their emails along these lines. Additionally, we know from outside reports that Gmail grew in popularity in between the time we originally collected data and when we conducted the second wave of data collection, while Yahoo, Hotmail and AOL declined in use (Creager, 2011; Dupre, 2014; Khan, 2015). More broadly, because people frequently use the same usernames repeatedly across accounts (Jacobsson Purewal, 2015), researchers may succeed in locating participants by pairing any usernames on file with the latest or most popular email or social networking providers.

Third, in cases in which we were unable to find active contact information for families by the means described above, about 4 months into re-recruitment, we attempted to contact parents through Facebook, Twitter, and LinkedIn, identifying profile pages connected with parents in 15 families. As per prior research (Masson et al., 2013), we occasionally searched through publicly available lists of parents' contacts on these networks to ensure we had identified the correct family (e.g., searching if a potential parent was connected to his/her spouse as identified on the Time 1 contact forms).

We debated creating clean study accounts vs. using our personal accounts to message participants, but ultimately decided to use our personal accounts for enhanced credibility (i.e., to come across like real people and not spammers; T. Rousse, personal communication, June 28, 2016). Accordingly, we suspected recruiters who were similar to the sample parents (i.e., fellow parents of school-age children) might be more successful recruiting in this manner, assuming study parents were able to infer such similarities from the public portions of researchers' profiles (see Ribisl et al., 1996 for a similar discussion about recruiter-participant similarities in the context of more traditional location methods). Nonetheless, in large part due to a lack of statistical power, our data do not provide clear evidence for or against this supposition: The youngest undergraduate researcher on our team received a response from 0 out of the 3 families she contacted via social media, the faculty member (who had children) received a response from 1 out of 8 families she contacted, but the doctoral student researcher (who had no children) received a response and scheduled 1 out of 3 families she contacted.

Despite searching across multiple social networking sites, we only successfully recruited (and scheduled) through Facebook, paralleling other recent non-prospective longitudinal studies that relied on social media for re-recruitment (Masson et al., 2013). These findings may reflect Facebook's popularity among middle-aged adults relative to other social networking sites at the time of data collection (Duggan et al., 2015). Interestingly, in other work, participants reported preferring to be contacted through Facebook over traditional mail or telephone because they felt Facebook was private and could conveniently be accessed by both smartphone or computer (Masson et al., 2013).

#### Searching for participants via paid online databases

Like others (e.g., Cotter et al., 2002), after exhausting the aforementioned free options, we used two fee-based contact information databases to search for 27 families. We paid $40 for access to PhoneDetective.com and $30 for access to Intelius.com. There are mixed opinions on the usefulness of paid contact information databases. Some prior studies have utilized such search engines early into their re-recruitment efforts and found them to be quite useful (e.g., Haggerty et al., 2008). Others, however, are more skeptical about their credibility (Cotter et al., 2002). Some paid search engines use marketing language that might give users unrealistic expectations about the quality of the results they can yield; these paid search engines may not provide much, if any, contact information that users could not find for free (Boswell, 2007). We did not begin using paid search engines until late into our recruitment process, after we had already drawn heavily from available free resources. This may be why we located so few participants via these two databases. Altogether, this suggests that researchers with limited resources may be successful re-recruiting with free resources and should wait to resort to paid databases until they are confident that have exhausted the data available for free.

#### Additional location strategies

Some additional search strategies addressed by other investigators may be useful when re-locating families, despite not being feasible for us. These include:

- Leveraging relationships with partner organizations (e.g., schools, after school programs) to facilitate continued data collection (e.g., Agrawal et al., 1978; Krajewski and Schneider, 2009; Lazorick et al., 2014). In some cases, organizations such as school districts may help re-locate original study participants (Tourangeau et al., 2005) or provide researchers useful contact information (Agrawal et al., 1978). These sorts of researcher-organization partnerships are common in prospective longitudinal studies with children (e.g., Tourangeau et al., 2005; Language Reading Research Consortium et al., 2016). Although policies differ from one school district to the next, the general consensus seems to be that for a school or similar organization to connect researchers with former participants, they (i.e., families) must have initially consented to future contact, as well as for the school district sharing their contact information (Ribisl et al., 1996). Nonetheless, even with these conditions in place, schools still would likely require submission to the school district research review board, which is a time-consuming process with no guarantee of additional participant recruitment information. Likewise, as children enter new grades and as staff and priorities fluctuate, new teachers and administrators would need to be convinced of a research study's importance, regardless of their predecessors' previous support (Language Reading Research Consortium et al., 2016). It also may be necessary to pay schools a fee to facilitate this form of collaboration. In some cases, it may be possible to obtain contact data from schools free-of-charge if a study benefits the district, for example, by providing data about a school-based program's effectiveness (P. Godard, personal communication, August 10, 2016). However, in other cases, school districts may charge researchers a fee regardless of educational relevance to cover the staff time invested in pulling the data (S. Dickson, personal communication, August 12, 2016). Some school districts may be more helpful if they have received a grant related to the study, or if the research activities otherwise appear to benefit the school or district (Language Reading Research Consortium et al., 2016).
- Visiting the neighborhoods participants lived in at the time of the original study (e.g., Ribisl et al., 1996; Haggerty et al., 2008). Because families with young children can develop attachments to their neighbors and first home, cumulative inertia (i.e., resistance to moving the longer a family stays in one location) sometimes sets in until additional life events impact the probability of moving (such as growing family size, change in jobs, desire for more space; Huff and Clark, 1978). As such, prior researchers have had modest success locating original study participants by visiting their former homes and neighborhoods, and reaching out to their friends and neighbors (e.g., Anderson et al., 2001; Haggerty et al., 2008). Studies with a primarily middle-SES sample may note particularly high levels of cumulative inertia and residential stability, as prior research indicates that those with average income or education levels are less likely to move than persons at the extremes (Abu-Lughod and Foley, 1960). Accordingly, visiting the neighborhoods participants lived in at Time 1 could be fruitful for teams working with certain populations, but should only be attempted in cases where researchers gained appropriate consent as described above.
- Posting local advertisements. Barakat-Haddad et al. (2009) located a small number (less than 1%) of children in their longitudinal study through advertisements in local newspapers. In rural, small, or tight-knit communities, local and grassroots outreach could provide another avenue for locating former participants.

#### Concluding notes on location

We believe all the location approaches described above complement one another, and indeed, others have suggested multiple recruitment outreach methods often work in tandem (Ribisl et al., 1996). Several parents who previously ignored our telephone calls responded positively to calls after receiving a written letter describing the study. Likewise, all the parents who responded to our recruitment attempts via text message had received calls from us first.

Our recruitment approach also evolved over time in a way that may have impacted results. For example, we did not begin sending postal mail to families until the third month of re-recruitment or contacting them via social media until the fourth month of recruitment, and did not begin to use paid databases until we felt we had exhausted free resources. We also made minor tweaks to our protocol over the course of re-recruitment. To illustrate, one father emailed us after receiving a re-recruitment letter and explained that it piqued his daughter's interest, at which point we began addressing letters to both parents and children. We had already sent letters to 33 families addressed just to parents, but from that point forward addressed letters to both parents and children, with 26 families receiving such letters. Although we successfully located two families using this parent-child address approach, it is unclear the extent to which this tweak affected our location rate given the small sample size. In contrast to these more fluid aspects of our location approach, we also used the same invariant series of steps when reaching out to parents on typical outreach days, privileging phone calls over other location approaches. Consequently, other teams of researchers may see recruitment rates vary relative to what we report in Table 1 based on variation in their re-recruitment protocols.

Experienced or committed research staff may be able to brainstorm additional ways to search for contact information specific to local communities or more efficiently leverage the latest search tools. As prior research suggests, more experienced researchers are often stronger recruiters in general (Sugden and Moulson, 2015). We found undergraduate volunteers often needed quite a bit of direction when searching to yield usable location data.

Throughout the location process, we maintained a detailed log of our communication attempts across platforms, somewhat aligned with recommendations from Cotter et al. (2002) and Ribisl et al. (1996). For efficiency's sake, we categorized phone and email contact information according to whether we had (a) confirmed its connection to a participant, (b) denied its connection to the participant (i.e., wrong or inactive phone number or email), (c) not yet tested it for connection, or (d) tested it but not received a definitive confirmation or denial. Though retaining this level of detail did not result in the most streamlined database, we found it necessary to avoid wasting time retesting communication avenues we had already deemed unhelpful. In additional columns in the database, we recorded the date and time of previous communication attempts, successful or not, keeping the most recent attempt on top for ease of determining when someone had last been contacted. This allowed us to diversify the timing of our attempts.

### Scheduling families for study participation

After (or in our case during) the location process, researchers scheduled participants for study appointments. In our follow-up study, we successfully collected Time 2 data from 101 families (83% of the located sample and 74% of the full original sample). The remaining located families either refused to participate in the Time 2 study (6 families; 5% of located families), missed or canceled appointments and were unresponsive to our attempts to reschedule (6 families; 5% of located families), or failed to ever schedule appointments before the conclusion of data collection (8 families; 7% of located families). Of the families we did test, it took on average 21.20 days (*SD* = 28.07) from first locating them to completing research sessions with them. Again, we encountered a great deal of variation in ease of scheduling, holding sessions with 40 families (40% of participating families) within 1 week of location, 41 (41%) within 1 month, and 20 (20%) after 1 month had past. Our data mirrors prior work; for example, Cotter et al. (2002) reported that about a third of their pediatric mental health clinic sample was easy to schedule, a sixth required multiple contact attempts before scheduling, and 7–8% refused to participate in some follow-up sessions. Table 1 provides a more detailed breakdown of how long it took us to schedule participants after locating them via each method described in the previous section. Among the 122 located families, those who scheduled and attended Time 2 appointments trended toward being less affluent than those we located but failed to schedule and collect data from [*t*_(24.10)_ = −1.76, *p* = 0.09], according to Time 1 data. However, no other fully or marginally significant differences emerged between these groups.

When reaching out to schedule appointments, we included language we thought might motivate busy families to make time for our study (Striano, 2016). For credibility, we referenced our university toward the beginning of most recruitment communications (Silva, 1990; Haggerty et al., 2008; Sugden and Moulson, 2015). As a potential appeal, we also explained the overarching study goal (Silva, 1990; Ribisl et al., 1996), which was to evaluate the long-term effectiveness of educational computer games created with funding from the U.S. government. We assumed this approach would speak to the parents in our sample, who had enrolled their children in an optional educational computer game intervention when children were in preschool or kindergarten. Later in our recruitment process, we began describing to parents roughly how many families had already participated in the Time 2 study. We intended for this to both make parents feel as if they were part of something large and important and to legitimize and normalize participation. Indeed, Rosenthal and Rosnow (1975) reported that people are more likely to volunteer for research studies when they believed such participation was normative (as cited in Bordens and Abbott, 2014). We also mentioned that participation would help the doctoral researcher conclude her dissertation project and graduate, to associate participating with an additional positive outcome. Evoking a personal connection and opportunity for parents to be helpful may explain why the doctoral student had slightly more success recruiting via social media than the other team members, although again, we can only speculate about this due to a lack of statistical power.

Furthermore, we provided parents and children each $20 for participating, and highlighted this in recruitment communications. This compensation seemed highly motivating to children but less so to parents. Minimum wage was about $10 per hour in the city where we collected our data, and the cost of living was higher than the national average. The relatively low incentive for this area may explain why some of the affluent families we located ultimately did not schedule and follow through with appointments. Other longitudinal work providing larger compensation yielded somewhat higher scheduling rates (e.g., Haggerty et al., 2008), although over-compensating families, especially lower-income families like many in our sample could be considered unethical or coercive (Hartmann, 1992; American Psychological Association, 2010).

Initially, we described to parents what we believed were salient aspects of the original study, attempting to trigger positive memories or loyalty to the intervention (see Ribisl et al., 1996; Cotter et al., 2002 for a discussion on the value of study loyalty and affiliation). However, it became clear to us over the course of data collection that many parents struggled to recall the Time 1 intervention because of the 6-year time lapse. Thirteen parents wrote in Time 2 questionnaires that they did not recall the Time 1 intervention or wrote comments clearly confusing our study with ones conducted by other groups on other topics. Similar difficulties also have been suggested in other longitudinal studies with long time gaps between waves of data collection (Barakat-Haddad et al., 2009). This may explain in part why the faculty member, who was the only Time 2 study team member involved in the original data collection, was not more successful recruiting via social media—weak personal connection (although again, a larger sample would be needed to more definitively support this conclusion). To further jog memory and enhance credibility, we began taking care to name the preschool or kindergarten where families were initially recruited about 6 weeks into Time 2 re-recruitment. As with the other tweaks made to our location strategy over the course of the study, it is unclear the extent to which this change impacted our success. We re-recruited most families prior to making this change, but this or the combination of messaging may have prompted a positive response from initially reluctant families. Nonetheless, memory-related recruitment issues may play less of a role in longitudinal studies with a shorter gap between waves of data collection.

Across all recruitment messaging, some parents seemed to extrapolate a sense that participating in the study would somehow provide children an academic enrichment opportunity, aid them as parents in better facilitating children's continued education, or otherwise abstractly improve children's education. Similarly, some parents said that they ultimately decided to participate in the study hoping it would help inspire their children to attend Northwestern University, where the research team was based. Since different appeals may speak to different families, it may be best to position a study as accruing a variety of tangible benefits (Sugden and Moulson, 2015; Striano, 2016).

Adhering to recommendations in prior methodological pieces, we provided participants a great deal of flexibility in terms of scheduling (Ribisl et al., 1996; Cotter et al., 2002). For convenience and comfort, we gave families the choice of participating in their homes (an option chosen by 45 families, 45% of participants), our lab space in a suburb just outside Chicago (22 families, 22% of participants), or local libraries with private study rooms (23 families, 23% of participants). It is likely that this flexibility increased participation rates, as logistical barriers such as inflexible work schedules, lack of transportation, or need for child care for younger children are known to impact the ability to retain low-income families in research (Duch, 2005). Additionally, 11 families (11%) who had moved out of the metropolitan area completed online surveys instead of participating in person (another strategy also recommended in other recent methodological reviews; e.g., Kalkhoff et al., 2014). This helped to address physical barriers found in other studies, such as where some participants were difficult to obtain data from due to physical constraints (e.g., military training, incarceration, and overseas relocation; Anderson et al., 2001). We accommodated most appointment time requests, excluding cases, for example, when families wanted to meet late at night or at local libraries during hours they were not open. As alluded to above, we tried to schedule appointments as soon after reaching participants as possible, ideally within 1 week of initially locating families (Ribisl et al., 1996; Kalkhoff et al., 2014). If we did not have much availability over the course of the following week, we held off attempting to contact parents until our schedule was more open rather than seeking appointments weeks in advance. This practice sought to minimize the window in which families could forget about appointments or experience other schedule changes.

Online and digital tools can further help with appointment management. We manually entered study appointments into a digital calendar shared by the research team, emailed parents event appointments that could be added to any personal digital calendars they maintained, and programmed these event appointments to send parents email and calendar pop-up reminders the night before or morning of their scheduled study sessions. Others have reported using digital interfaces such as YouCanBook.Me to allow participants to privately and relatively independently sign-up for and, if need be, reschedule study appointments, choosing among several session times researchers make available (Kalkhoff et al., 2014). These interfaces also can send automated appointment reminders in advance of sessions (Kalkhoff et al., 2014).

## Confirming appointments

As with all developmental research, the last step in our re-recruitment process was to confirm appointments with parents, making it clear we would be happy to reschedule if need be (Striano, 2016). Initially, we confirmed appointments exclusively by phone call and email, aligned with methodological recommendations elsewhere (Kalkhoff et al., 2014), but later into recruitment, began calling, emailing, or texting parents, depending on their preferences.

Even though only a small number of parents responded to our early locating/scheduling efforts by text message (see Table 1), this communication channel worked well for confirmation, perhaps because families perceived text messaging to be a less formal means of communicating. Four dyads (11% of in-person appointments scheduled at the time) missed their appointments before we started texting parents, but this only occurred once (2% of in-person appointments) after we introduced texting (and this one appointment was missed due to an unfortunate family emergency). Parents who needed to cancel or reschedule appointments seemed more comfortable doing so via text rather than over the phone or through email. Moreover, if researchers suspected a participant might forget about the appointment after official confirmation, they would send casual “on my way” text messages to prompt parents' memories. We each used our personal phones for such purposes, although it might have been wiser to buy a study-specific cell phone or set up a Google Voice account that researchers could share without compromising privacy (M. Smith, personal communication, April 27, 2016). Some of the digital study appointment management tools described in the previous section can automatically send confirmation emails or text messages, and may allow parents to reschedule appointments without needing to interface with a researcher (Kalkhoff et al., 2014).

## Conclusion

Across developmental sub-fields—from basic cognition (e.g., Lauer and Lourenco, 2016) and language development (e.g., Can et al., 2013) to mental (e.g., Agrawal et al., 1978) and physical health (e.g., Fein et al., 2014) to applied interventions targeting children (e.g., Campbell et al., 2012) and their families (e.g., Huston et al., 2005)—researchers are engaging in longitudinal work, re-recruiting families who participated in one study to gain a better understanding of children's developmental trajectories. Frequently, research teams do not decide to begin embarking on this work until several months or years after an original study has concluded. Our work demonstrates the feasibility of re-recruiting sizeable numbers of urban families after an extended gap in communication with limited financial resources. Future developmental research teams should be able to achieve high follow-up rates by (a) setting up initial studies in which parents provide detailed contact information, including contact information for multiple caregivers, and consent for later waves of research, (b) searching across a variety of sources to locate participants, e.g., people-centric search engines, social media, etc., (c) writing multifaceted scheduling phone/email/mailing scripts highlighting the study's value, and (d) confirming appointments in a way that conveys a casual tone that makes parents feel comfortable, even if they need to reschedule. Many of these strategies may likewise enhance the recruitment process even for cross-sectional research. Developmental researchers also may wish to consult Table 1 to help inform timelines as they plan, keeping in mind that success rates and timing may vary based upon the order in which researchers employ each location strategy.

Relative to prior research, we had more success re-recruiting with free tools such as people-centric search engines, and less success using paid and other tools. Differences between our study and others may be attributable in part to advances in modern technology and our efforts to leverage popular technological services. Because parents are increasingly maintaining the same cell phone numbers, especially among high- and middle-income populations, even after they move (Dost and McGeeney, 2016) and because of the existence of a plethora of free people-centric search engines, we had more success using originally provided telephone numbers and free search engines than researchers reported previously. We similarly found text messaging, which is currently very popular in the U.S. (Duggan, 2013), to be helpful in ensuring participants maintained their appointments or felt comfortable rescheduling if necessary. Indeed, even researchers conducting cross-sectional work may wish to consider incorporating text messages into their appointment confirmation protocols. We only saw limited success locating participants via social media or paid databases, somewhat contrasting prior research where paid databases were more effective (e.g., Haggerty et al., 2008). However, we suspect our location findings would have varied had we engaged the various search methods in a different order.

Technology-related advances aside, our study also reinforces the value of strategies others have suggested to schedule participants and calls into question assumptions about the ease of scheduling particular groups of participants. Our experiences underscore the importance of collecting detailed contact information during an initial study (Ribisl et al., 1996) and using recruitment (or re-recruitment) phone/email/mail scripts that make the university affiliation and study goals clear (Sugden and Moulson, 2015). Such re-recruitment messages may be especially valuable in cases where researchers are focused on academic, prosocial, or other potentially positive outcomes. Moreover, emphasizing the university and study aims may even be more worthwhile than reminding families of the particulars of the original study. Like others (see Ribisl et al., 1996), we also believe our re-recruitment success is in part attributable to the fact that we planned our Time 2 study activities in a way that allowed us to conduct research in a variety of settings (i.e., lab, library, home). However, we recognize such flexibility may not be feasible for all development sub-domains, such as when researchers are interested in collecting neurological data (e.g., Schwartz et al., 2003).

Like some studies, we had somewhat more success locating families with higher-incomes, as well as Caucasian families (e.g., Fein et al., 2014). At least for our study, we suspect these findings may both trace back to stability issues related to family income, rather than anything cultural. Caucasian families in our study were more affluent than the rest of the sample [*t*_(52.93)_ = 7.97, *p* < 0.001], a pattern that bears out nationally in the U.S. (Wilson, 2015). Low-income families, regardless of race-ethnicity, are more likely to experience disruptions in phone service, changes to cell phone numbers (Ahlers-Schmidt et al., 2012), and physical relocation over time (Abu-Lughod and Foley, 1960), factors which in turn might make them particularly challenging to locate. However, mirroring other findings that contradict our location results (e.g., Silva, 1990), we were arguably *less* successful *scheduling* higher-income participants. To increase scheduling rates, those working with primarily affluent samples may wish to provide larger monetary compensation when doing so would not be coercive.

Rosenthal and Rosnow (1975) suggest researchers also may want to place an even greater emphasis on situational factors that improve participation, such as the normative nature of participation, state the theoretical as well as the practical importance of the study, and make clear the manner in which their participation is relevant (as cited in Bordens and Abbott, 2014). In addition, stressing the ease of participation and having the request made by a trustworthy person of as high of status as possible can help to increase interest and appeal (Ribisl et al., 1996).

As is typical in longitudinal research (Ribisl et al., 1996), we attempted to re-recruit all the original families and used all the allotted appointment time during each research session to gain information about children's present, study-specific functioning. Consequently, we did not formally survey parents about their perceptions of the re-recruitment experience or experimentally compare the effectiveness of different recruitment strategies (which might have resulted in us losing participants assigned to less successful recruitment strategy conditions). Moreover, we refined our recruitment approach over the course of data collection, as is common in research of this nature (see Ribisl et al., 1996). Future researchers with larger initial samples or more time and financial resources should consider formally testing some of the assumptions in this article; such work would address important gaps in the methodological literature.

Despite the aforementioned limitations, we hope our efforts can guide other developmental scientists interested in conducting longitudinal research. Moreover, some of these strategies may even positively impact recruitment for cross-sectional studies. Given the power longitudinal studies have to clarify developmental trajectories (Nicholson et al., 2002) and provide compelling accountability evidence for interventions (e.g., Barnett, 2013), and given the growing interest in work of this nature (e.g., Wartella et al., 2016) it is important developmental scientists feel capable of re-recruiting sufficiently large samples, even with limited resources and even when they decide to begin such work long after the conclusion of a particular study.

## Ethics statement

This study was carried out in accordance with the recommendations of our Universities & Institutional Review Boards (IRBs) with written informed consent from all parents and verbal or written assent from all children. All parents gave written informed consent and all children gave verbal or written assent in accordance with the Declaration of Helsinki. The protocol was approved by our IRBs.

## Author contributions

All authors listed have made a substantial, direct and intellectual contribution to the work, and approved it for publication.

### Conflict of interest statement

KS is the owner and President of KL Media Research. The other authors declare that the research was conducted in the absence of any commercial or financial relationships that could be construed as a potential conflict of interest.
